# Examining the Connectorship Scale: Factor Structure and Correlations With Self-Efficacy and Extraversion

**DOI:** 10.1177/00332941241248597

**Published:** 2024-04-23

**Authors:** Hardi Vadher, Kristi Baerg MacDonald, Sarah Ross, Julie Aitken Schermer

**Affiliations:** Psychology Department, 6221 The University of Western Ontario, London, ON, Canada; Psychology Department, The University of Western Ontario, London, ON, Canada; Management and Organizational Studies, The University of Western Ontario, London, ON, Canada; Psychology Department, The University of Western Ontario, London, ON, Canada; Management and Organizational Studies, The University of Western Ontario, London, ON, Canada

**Keywords:** Self-efficacy, extraversion, connectorship, engaged leadership, leadership

## Abstract

The Connectorship Scale was designed to assess how leaders connect with their followers and is described to measure eight dimensions: social interactivity, dependability, positive communication, presenting oneself, storytelling ability, belief in networking, tangible introduction, and belief in the importance of online networking. This study explores the scale properties and confirmatory factor analyses (CFA) of the Connectorship Scale and examines how the scale scores correlate with self-efficacy and extraversion based on responses from 454 (52% women) adult business students. The internal consistency estimates suggested that one of the subscales, positive communication, was unreliable; we therefore excluded that subscale from further analyses. A CFA of the seven-factor model suggested good fit once two pairs of error terms were allowed to correlate. Self-efficacy and all facets of extraversion positively correlated with six of the seven connectorship subscales, the exception being the tangible introduction scale. The results raise concern about the positive communication subscale from the Connectorship Scale but do support the use of the other seven subscales for research about engaged and effective leadership.

## Introduction

The purpose of the present study is to examine the Connectorship Scale (Dastmalchian et al., 2016). Connectorship is defined as the quality that binds leadership and networking and involves creating meaningful relationships with followers (Dastmalchian et al., 2016). In their scale, Dastmalchian et al. (2016) report that connectorship is composed of eight facets, including: interactivity, dependability, positive communication, presenting oneself, storytelling, belief in networking, tangible introduction, and online networking. (Dastmalchian et al., 2019). The goal of this study is to assess the Connectorship Scale (Dastmalchian et al., 2016) in terms of determining internal consistency estimates of the sub-scales, testing the proposed factorial structure of the measure using confirmatory factor analysis, and assessing convergent validity by computing the associations with two traits that have been demonstrated to be positively associated with effective leadership, specifically self-efficacy and extraversion (Ensari et al., 2011). Because leadership assessment is important for both research and applied settings, understanding if the proposed characteristics of a leadership scale are supported with an independent sample is essential knowledge. Following, the present study is possibly the first independent assessment of the Connectorship Scale.

### Connectorship Defined

Leadership can be conceptualized as the use of power and influence to set expectations and direct the activities of followers to achieve goals (Colquitt et al., 2016). Leadership is important for establishing direction, aligning resources, and motivating followers to maximize efficiency and meet objectives (Campos et al., 2020; Kotterman, 2006). Dastmalchian and colleagues (2019) argue that to be an effective leader, an individual must be an engaged leader who is capable of leading, forming social networks, and connecting to people within those networks.

Engaged leadership includes traditional leadership skills, the capability to network, and the ability to connect with others (Dastmalchian et al., 2019). Connectorship, one component of engaged leadership, is about building stronger relationships between leaders and followers. Connectorship binds the elements of a being able to lead and network, and therefore is a construct of particular importance. Research by Schafer (2010) suggests that ineffective leaders lack interpersonal skills, possess poor communication, and have an inferior work ethic. If a leader is unable to connect well with their followers, the leader is likely to be ineffective. Lipman-Blumen (1992) investigated connective leadership, a construct closely related to connectorship, and proposed that the ability to facilitate connections with one’s followers is a necessary skill to being an effective leader. Qualities such as attentive listening, exhibiting positive body language, engaging in a mutual exchange of information, and following up on new connections are associated with one’s ability to connect with others effectively (Chatman et al., 2020; Dastmalchian et al., 2016). Further, Robinson (2016) indicated that a connective leader is one who is flexible in leadership styles and can adapt to environmental changes. By possessing the quality to adapt to their followers’ needs, leaders can better connect and subsequently, be more effective (Robinson, 2016). If a leader is not able to remain involved with their pre-established connections, make active use of these resources, or build new connections, then they may not succeed in being an engaged leader. Hence connectorship is a necessary component of the engaged leadership framework (Dastmalchian et al., 2016).

Dastmalchian et al. (2016) developed the Connectorship Scale based on a qualitative study that consisted of 18 in-depth interviews with Canadian business leaders who described their perceptions of connectorship. The original 93-item scale (Dastmalchian et al., 2016) was intended to measure five dimensions of connectorship (conversation skills, sociability, connectivity, presence, and support), but an exploratory factor analysis (EFA) using principal component analysis with varimax rotation revealed eight factors.

The eight-factor structure was supported by a confirmatory factor analysis (CFA) using a sample of managers from various industries and organizations across Canada (Dastmalchian et al., 2016). The scale authors eliminated items with poor fit, resulting in a final questionnaire with eight conceptually distinct dimensions and 28 items (see Dastmalchian et al., 2016, p. 413 for the item content). In the present study, we examine the factor structure of the scale to evaluate whether the eight factors present a good fit with the current data. Results in agreement with the eight-factor structure would provide support for the Connectorship Scale and its use in measuring a component of engaged leadership.

### Self-Efficacy and Extraversion

In their meta-analysis, Ensari et al. (2011) found that both self-efficacy and extraversion are positively associated with leadership emergence and that extraversion was one of the strongest personality predictors of leadership. If self-efficacy and extraversion predict leadership, then positive correlations between self-efficacy and extraversion with connectorship scale scores would add to the construct validity of the scale. This question is examined in the present study.

Self-efficacy is the belief in one’s ability to successfully meet a given situation’s demands (Bandura, 1977). Machida and Schaubroeck (2011) discovered that self-efficacy plays an instrumental role in a leader’s development. If an individual believes in their capacity to make a consistent effort to grow as a leader, their persistence to enhance their leadership skills may translate into becoming a more effective leader (Erozkan, 2014; Forret & Dougherty, 2001). Further, Chen et al. (2006) suggested that to be a successful leader, an individual requires high self-efficacy in their ability to plan, organize, and perform those plans effectively with their followers. Ramchunder and Martins (2014) found a strong positive relationship between self-efficacy and leadership in a policing context and Tian (2024) reported a positive association between transformational leadership and self-efficacy in a large sample of teachers. Sun et al. (2024) reported across three studies that self-efficacy was associated with ethical leadership and knowledge sharing. Similarly, Khorakian and Sharifirad (2019) reported a positive correlation between self-efficacy and leader-member exchange among employees. Walumbwa et al. (2011) reported that self-efficacy is positively linked to ethical leadership. Based on this research, if a positive correlation is found between self-efficacy and the connectorship scale scores, this evidence may suggest some support for convergent validity for the scale.

Extraversion reflects individual differences in the tendencies to experience and exhibit gregariousness and warmth in social situations and includes being active, excitement-seeking, having positive affect, and displaying assertive behaviour (Wilt & Revelle, 2017). Silverthorne (2001) found that effective leaders are more extraverted than ineffective leaders, but a meta-analysis conducted by Bass (1990) revealed an inconclusive relationship between extraversion and leadership. In contrast, a meta-analysis by Judge et al. (2002) suggested that extraversion has a strong and consistent relationship with leadership across study settings and leadership criteria (e.g., leadership emergence and effectiveness). At the domain level, Karlsen and Langvik (2021) reported that leaders who self-reported higher extraversion levels were rated more positively by followers. Likewise, a meta-analysis by Do and Minbashian (2014) also found extraversion, specifically gregariousness and warmth, to be strong predictors of effective leadership.

Lee et al.’s (2008) study revealed a moderately strong relationship between extraversion and social connectedness, a concept similar to connectorship. While some facets of extraversion are more distinctly related to leadership than others, each facet of extraversion may have the potential to be positively related to how connected a leader and follower are to each other (Lee et al., 2008). Hence, if an individual is more extraverted, it may be that they are likely to score higher on connectorship. If a positive correlation is found between extraversion and connectorship, this relationship may add to the convergent validity of the scale.

### The Present Study

To our knowledge, no previous studies have both examined the Connectorship Scale (Dastmalchian et al., 2016) properties, independent of the studies conducted by the scale authors, and examined the relationships with self-efficacy and extraversion. The present study examines the internal consistency estimates of the eight subscales as well as the factor structure, using CFA of the eight-factor solution. In addition, the correlations between the connectorship scores with self-efficacy and extraversion are tested, with expected positive correlations.

## Method

### Participants and Procedure

Undergraduate business management students (*N* = 454 students) participated in the present study for a research credit which was granted regardless of individuals completing the scales. Participants who provided complete data included 235 women, 218 men, and one case preferred not to disclose. The age of the participants ranged from 17 to 42 years, with a mean age of 18.35 (*SD* = 1.73). Participants provided informed consent and for those who chose to, completed questions about their age and assigned sex at birth as well as a set of self-report questionnaires described below. The study received institutional ethics approval (REB# 116,572) and was performed in accordance with the ethical standards as set forth in the 1964 Declaration of Helsinki and its later amendments as well as the Canadian Tri-Council Policy Ethical Conduct for Research Involving Humans (TCPS 2). The data is available by contacting the submitting author.

## Measures

### General Self-Efficacy Scale

The General Self-Efficacy Scale (Schwarzer & Jerusalem, 1995) was used to measure a general sense of perceived self-efficacy. This unidimensional scale consists of 10 self-report items, rated on a 4-point Likert scale (1 = *not at all true* to 4 = *exactly true*). Participants were asked the extent to which they agreed with statements such as, “I can always manage to solve difficult problems if I try hard enough.” In the present study, the Cronbach’s alpha was 0.86.

### Neuroticism-Extraversion-Openness Personality Inventory-Revised (NEO-PI-R)

The NEO-PI-R assesses Neuroticism, Extraversion, Openness to Experience, Agreeableness, and Conscientiousness (Costa & McCrae, 1992). The full NEO-PI-R includes 240 items (48 items per trait) responded to with a 5-point Likert scale (1 = *strongly disagree* to 5 = *strongly agree*). In the present study, only the extraversion scale was used, measuring six facets: warmth, gregariousness, assertiveness, activity, excitement-seeking, and positive emotions. The extraversion scale consists of 48 self-report items, wherein participants are asked to rate the extent to which they agree with statements such as, “I really like most people I meet” (for the warmth facet) and, “I have often been a leader of groups I have belonged to” (for the assertiveness facet). The coefficient alpha for the facets ranged from 0.61 for activity to 0.78 for warmth. The coefficient alpha for the complete scale was 0.89.

### Connectorship Scale

The Connectorship Scale (Dastmalchian et al., 2016) was used to measure an individual’s capability to connect with others in their social network. Factors within this scale are interactivity (10 items), dependability (4 items), positive communication (2 items), presenting oneself (3 items), storytelling (2 items), belief in networking (3 items), tangible introduction (2 items), and online networking (2 items). The scale consists of 28 self-report items, rated on a 7-point Likert scale (1 = *strongly disagree* to 7 = *strongly agree*). Participants were asked to rate the extent to which they agree with various statements about themselves such as, “I have good social etiquette and manners” (for the interactivity factor) and, “My online network is effective in keeping me connected with people” (for the online networking factor). Internal consistency of the positive communication factor was very low (α = .33); as such, we did not include this factor in the analyses. The coefficient alpha for the remaining factor scales were 0.82 for interactivity, 0.78 for dependability, 0.88 for presenting oneself, 0.74 for storytelling, 0.67 for belief in networking, 0.66 for tangible introduction, and 0.78 for online networking. The coefficient alpha for the complete scale without the positive communication items was 0.88. Each scale had skewness values less than ±1 and kurtosis values less than ±1.

## Results

### Confirmatory factor Analysis

The confirmatory factor analysis (CFA) with maximum likelihood (ML) estimation was conducted using R version 4.0.2 (R Core Team, 2020), primarily using the lavaan package (Rosseel, 2012). Missing data was also treated with the maximum likelihood method. The factors for the CFA were defined by the division of items described by Dastmalchian et al. (2016), hypothesized to be interactivity, dependability, presenting oneself, storytelling, belief in networking, tangible introduction, and online networking. Dastmalchian et al. (2016) also included positive communication, but as described earlier, this scale was not used due to very low internal consistency.

The original model (Model 1), defined by Dastmalchian et al. (2016), showed marginal fit (see Table 1 for fit indices). Based on information from modification indices, we examined the scales and ran the analysis again, allowing correlated error terms for items 13 (“I am honest”) and 14 (“I am truthful”) as Model 2. The fit improved, and we ran one more model, Model 3, allowing items 2 (“I have good social etiquette and manners”) and 4 (“I am aware of and sensitive to the feelings of others”) to correlate. Model 3 showed marginal to good fit. The chi-square (χ^2^_(276)_ = 708.99, *p* < .001) was significant, which is not unusual in larger samples. Other fit indices show a good fit: comparative fit index (CFI = .91), and standardized root-mean-square residual (SRMR = .05; Hu & Bentler, 1999). The root mean square error of approximation (RMSEA = .06) was an approximate fit (MacCallum et al., 1996). Standardized factor loadings, listed in Table 2, were mostly strong, with all but one loading >.35 onto the hypothesized factor. Item 10, “I am concerned with how people interact” did not load highly onto the interactivity factor, but further examination of the modification indices, as well as theoretical reasoning, do not suggest that the item would be more appropriate on a different factor or that cross-loading would improve fit significantly. The fit of Model 3 is not as close as the fit initially found by Dastmalchian et al. (2016); however, their methodology was such that their initial model contained more items and items were deleted to generate better model fit.

**Table 1.** Summary of Original Model and Two Model Modifications.

Model	Summary of modification	*X*^2^ (*df*)	RMSEA (CI)	CFI	SRMR
Model 1: Original	N/A	941.97 (278) *p* < .001	.07 (.07–.08)	.85	.08
Model 2: Modification 1	Correlated error terms for items 13 and 14	733,53 (277) *p* < .001	.06 (.06–.07)	.90	.06
Model 3: Modification 2	Model 2 with correlated error terms for items 2 and 4	708.99 (276) *p* < .001	.06 (.05–.06)	.91	.05

**Table 2.** Standardized Factor Loadings for Connectorship Scales.

	Interactivity	Dependability	Presenting oneself	Storytelling	Belief in networking	Tangible introduction	Online networking
Q1	.64						
Q2	.64						
Q3	.70						
Q4	.45						
Q5	.74						
Q6	.63						
Q7	.56						
Q8	.57						
Q9	.44						
Q10	.17						
Q11		.73					
Q12		.82					
Q13		.51					
Q14		.49					
Q17			.86				
Q18			.95				
Q19			.73				
Q20				.77			
Q21				.76			
Q22					.81		
Q23					.77		
Q24					.39		
Q25						.55	
Q26						.89	
Q27							.82
Q28							.78

Note. Items are in the order as presented on page 413 by Dastmalchian et al. (2016).

### Correlational Analyses

To test the hypotheses that self-efficacy and extraversion would positively correlate with connectorship, Pearson correlations were conducted (see Table 3). The prediction of a positive association between self-efficacy and connectorship was supported in that moderate positive correlations were found. The prediction that extraversion would correlate positively with connectorship was generally supported although the correlations between the tangible introduction connectorship scale with the extraversion scale and facets were nonsignificant. Table 4 lists the inter-scale correlations for the Connectorship scales. Although the correlations were small to moderately large and positive, interestingly, the tangible introduction scale only had a significant correlation with the on-line networking scale.

**Table 3.** Correlations between the General Self-Efficacy, NEO-PI-R Extraversion, and Connectorship Scales.

Scale	1	2	3	4	5	6	7	8
GSE scale								
1. Self-efficacy								
NEO-PI-R – Extraversion scale								
2. Extraversion total	0.44*							
3. Warmth	0.26*	0.74*						
4. Gregarious	0.22*	0.77*	0.58*					
5. Assertive	0.51*	0.66*	0.29*	0.36*				
6. Activity	0.36*	0.63*	0.27*	0.31*	0.50*			
7. Excitement	0.36*	0.69*	0.32*	0.47*	0.35*	0.30*		
8. Pos emotions	0.16	0.70*	0.56*	0.44*	0.27*	0.30*	0.34*	
Connectorship scales								
9. Interactivity	0.52*	0.61*	0.46*	0.40*	0.51*	0.34*	0.44*	0.37*
10. Dependability	0.33*	0.30*	0.25*	0.14	0.23*	0.20*	0.21*	0.20*
11. Presenting oneself	0.40*	0.47*	0.26*	0.28*	0.60*	0.35*	0.27*	0.23*
12. Storytelling	0.27*	0.37*	0.23*	0.16	0.39*	0.27*	0.25*	0.23*
13. Belief in networking	0.28*	0.33*	0.23*	0.22*	0.31*	0.20*	0.22*	0.21*
14. Tangible introduction	0.13	0.05	−0.01	−0.02	0.11	0.15	0.01	−0.05
15. Online networking	0.21*	0.33*	0.19*	0.25*	0.27*	0.23*	0.24	0.18*
Mean	30.87	166.71	30.63	27.06	25.60	25.51	29.46	28.34
*(SD)*	(4.31)	(20.56)	(4.88)	(5.34)	(4.86)	(4.27)	(5.31)	(4.93)

Notes: *p < .001; two-tailed; GSE = General Self-Efficacy Scale (Schwarzer & Jerusalem, 1995); NEO-PI-R = Neuroticism, Extraversion, Openness Personality Inventory-Revised (Costa & McCrae, 1992); Connectorship Scale (Dastmalchian et al., 2016).

**Table 4.** Correlations between the Connectorship Scales.

Scale	1	2	3	4	5	6	7
Connectorship scales							
1. Interactivity							
2. Dependability	0.47*						
3. Presenting oneself	0.49*	0.24*					
4. Storytelling	0.39*	0.14	0.36*				
5. Belief in networking	0.42*	0.23*	0.37*	0.32*			
6. Tangible introduction	0.13	0.13	0.14	0.11	0.10		
7. Online networking	0.32*	0.16*	0.27*	0.25*	0.46*	0.33*	
Mean	55.58	24.06	15.33	9.11	17.17	7.35	10.41
*(SD)*	(7.59)	(3.15)	(3.74)	(2.71)	(2.75)	(2.64)	(2.45)

Notes: *p < .001; two-tailed; Connectorship Scales (Dastmalchian et al., 2016).

## Discussion

The current study examined both the scale properties and the factor structure proposed by Dastmalchian et al. (2016) for their eight-factor Connectorship scale. The positive communication subscale did not have adequate internal consistency and is likely not a useful part of the larger scale. Furthermore, one of the items on the interactivity subscale which identifies whether a rater is concerned with others’ interactions did not fit well, suggesting that the use of this item should be examined before including in future research with the scale. Less than strong results were found with the tangible introduction scale. The tangible introduction scale consists of two items and each specifically addresses business cards (“I always carry enough business cards” and “I am comfortable giving out my business cards”). These results may have been because students completed the scale items, and they may not be used to interacting with or using business cards. The results may also reflect a decrease in the overall use of business cards with an increase in social networking sites and the exchange of emails through mobile devices when networking.

Self-efficacy correlated positively with connectorship (although the correlation with the tangible introduction scale was nonsignificant), supporting our prediction based on past findings (Ensari et al., 2011) and aligns with research that found positive associations between self-efficacy and leadership (Ramchunder & Martins, 2014; Walumbwa et al., 2011). Those who believe themselves to be capable in meeting situational demands also are likely to believe in their ability to connect with others. Further, positive correlations between extraversion and connectorship were found (except for the tangible introduction scale). In general, these results are consistent with prior research that suggested a positive link exists between leadership and extraversion (Judge et al., 2002; Silverthorne, 2001), suggesting that extraverted individuals easily connect with others. For both sets of correlations, the results do support, to some degree, the convergent validity of the scale.

### Limitations and Future Directions

A key limitation of this study was the lack of workplace data. Evaluating connectorship in an applied setting would add considerable knowledge about individual traits that make an engaged leader. As hierarchical structures are more common in organizational settings (in comparison with the present study’s student sample), a stronger link among the variables of self-efficacy, extraversion, and connectorship may have been discovered in a workplace context, where leader-follower roles are better defined (e.g., clear management-subordinate roles) compared to student leader-follower relationships. Additionally, because connectorship involves interacting with people (Dastmalchian et al., 2016), it may be beneficial for future researchers to study this variable in a group setting in the workplace. The large sample of business management students enabled the results to be generalizable to other business students. The generalizability of this research would be furthered if similar research were conducted with other undergraduate samples or better yet, among employees from various domains (e.g., engineering, general science) to understand connectorship across different fields.

The results are also limited by the cross-sectional and correlational nature of the study. Although personality is relatively consistent across time for adults, less is known about people’s ability to connect with others. As such, studying the connectorship construct at differing ages for men and women and in different occupations using longitudinal data may provide additional insight on this matter. The correlational nature of the data prevents us from concluding with certainty that individuals scoring high on self-efficacy and extraversion are high on connectorship, and subsequently, are engaged leaders.

Further, only two individual differences – self-efficacy and extraversion – were evaluated in relation to connectorship. To understand additional traits that make an engaged leader, future research should consider looking at the rest of the Big Five personality traits (agreeableness, openness, conscientiousness) in relation to connectorship. Future researchers should also consider identifying whether connectorship may be a subcomponent of other sub-categories of leadership (e.g., transformational leadership). Additionally, individual difference variables in connecting with others should be examined in future research. For example, Mitchell et al. (2022) reported that extraversion is a stronger predictor of leadership for those with higher levels of communication skills. Unfortunately, the positive communication sub-scale of the Connectorship Scale demonstrated low internal consistency in this sample. Possibly including a communication measure would add to our understanding of how personality is associated with connecting with others through communication abilities.

## Conclusion

The purpose of this study was to examine the scale properties and correlations with self-efficacy and extraversion with the Connectorship Scale (Dastmalchian et al., 2016). Results revealed that seven of the eight scales were reliable (all except the positive communication scale), that an item on the interactivity scale did not fit well with the factor, and that although the other connectorship scales correlated positively with self-efficacy and extraversion, the tangible introduction scale did not correlate significantly. We suggest that future research with the Connectorship Scale first examine the scale properties before conducting further analyses with other variables as it may be the case that one (communication) and possibly two (tangible introduction) scales require removal. It may also be the case that the tangible introduction scale may require updating/rewriting before used in research or applied settings.

## Data Availability

Data is available from the corresponding author upon request.
